# Applying learning health systems thinking in codeveloping integrated tuberculosis interventions in the contexts of COVID-19

**DOI:** 10.1136/bmjgh-2022-009567

**Published:** 2022-10-31

**Authors:** André Janse van Rensburg, Inge Petersen, Ajibola Awotiwon, Max Oscar Bachmann, Robyn Curran, Jamie Murdoch, Christy Joy Ras, Lara Fairall

**Affiliations:** 1 Centre for Rural Health, University of KwaZulu-Natal College of Health Sciences, Durban, KwaZulu-Natal, South Africa; 2 Centre for Health Systems Research & Development, University of the Free State Faculty of Humanities, Bloemfontein, South Africa; 3 Institute of Global Health, University College London, London, UK; 4 Knowledge Translation Unit, Department of Medicine, University of Cape Town, Cape Town, Western Cape, South Africa; 5 Norwich Medical School, University of East Anglia Faculty of Medicine and Health Sciences, Norwich, UK; 6 School of Life Course and Population Sciences, King's College, London, UK; 7 King’s Global Health Institute, King's College, London, UK

**Keywords:** COVID-19, health systems, tuberculosis, health services research, screening

## Abstract

The COVID-19 pandemic reversed much of global progress made in combatting tuberculosis, with South Africa experiencing one of the largest impacts on tuberculosis detection. The aim of this paper is to share our experiences in applying learning health systems (LHS) thinking to the codevelopment of an intervention improving an integrated response to COVID-19 and tuberculosis in a South African district. A sequential partially mixed-methods study was undertaken between 2018 and 2021 in the district of Amajuba in KwaZulu-Natal. Here, we report on the formulation of a Theory of Change, codesigning and refining proposed interventions, and piloting and evaluating codesigned interventions in primary healthcare facilities, through an LHS lens. Following the establishment and formalisation of a district Learning Community, diagnostic work and a codevelopment of a theory of change, intervention packages tailored according to pandemic lockdowns were developed, piloted and scaled up. This process illustrates how a community of learning can generate more responsive, localised interventions, and suggests that the establishment of a shared space of research governance can provide a degree of resilience to facilitate adaption to external shocks. Four main lessons have been gleaned from our experience in adopting an LHS approach in a South African district, which are (1) the importance of building and sustaining relationships, (2) the utility of colearning, coproduction and adaptive capacity, (3) the centrality of theory-driven systems strengthening and (4) reflections on LHS as a framework.

**Summary box**

- There is increasing evidence for the utility of collaboration and coproduction in research, especially in low-income to middle-income countries.
- There are limited described adoptions of coproduced interventions in the area of tuberculosis (TB) prevention and treatment, particularly in terms of its relations to mental health and COVID-19.
- The application of a learning health systems (LHS) approach in a South African district illustrates how a community of learning can generate more responsive, localised interventions.
- The use of theory of change further helps to strategically steer health systems strengthening for integrated, person-centred TB, mental health and COVID-19 care, within the broader frame of a cyclical LHS approach.

## Background

The COVID-19 pandemic has highlighted the critical need for health systems resilience.^1^ This has particularly been the case among low-income and-middle income countries (LMICs), where the consequences are yet to be fully comprehended and understood.^2–4^ COVID-19 and tuberculosis (TB) are both primarily airborne infections and thus share similar prevention interventions in relation to wearing of masks, social distancing and the promotion of well-ventilated spaces, as well as screening questions. It is unclear to what extent the current pandemic will affect the reaching of global TB targets, as set out in the Sustainable Development Goals, the End TB Strategy and also in the political declaration of the United Nations (UN) high-level meeting on TB.^5^ It has been estimated that the current pandemic may reverse much of the progress made in combatting TB, with a global estimated increase of 0.2–0.4 million TB deaths in 2020.^6^ In contexts where an estimated 10 million people contracted TB in 2019, of whom the vast majority were in the Global South,^6^ the widespread emphasis on non-pharmaceutical interventions to combat the transmission of COVID-19 provides a valuable scaffolding opportunity for strengthening TB transmission prevention.^7^

South Africa, with 3.6% of the global TB burden, is considered one of the eight highest TB burden countries. Its 2019 incidence rate of 615 cases per 100 000 (compared with 177 per 100 000 average among high burden countries), along with substantial mortality rates, continue to present a huge burden on the health system.^6^ Moreover, many TB cases remain undetected in community settings, where two-thirds of those with positive symptoms often do not seek care, and one quarter do not regard TB as a concerning illness.^8^ These dynamics have been significantly aggravated by COVID-19—as of August 2022, more than 4 million of its 60 million population have been infected, with more than 102 000 deaths.^9^ Further, excess mortality figures suggest that 326 600 have died between May 2020 and August 2022.^10^ There were early signs that the pandemic disrupted the TB programme. There has been a 21% reduction in global TB cases from 2019 to 2020, with COVID-19 substantially hindering detection processes—South Africa being one of the countries experiencing the largest impact on TB detection^11^ Between March and June 2020, during the brunt of the first COVID-19 wave and its associated lockdowns, monthly TB notifications fell by more than 50%.^6^. People either could not visit primary healthcare (PHC) clinics due to lockdown conditions and lack of access due to public transportation restrictions, or due to widespread fears of infection.^12^ There was also increasing competition for resources that both TB and COVID-19 response efforts shared, including masks and access to GeneXpert diagnostic services.^13^ There was a 19.2% decline in numbers of PHC screenings for TB between 2019 and 2020, with an 18% decline in GeneXpert tests conducted during the same period.^14^ The number of GeneXpert positive tests declined by 33% between the lockdown period of February to May 2020, but rapidly returned to previous levels following a lifting of restrictions—this suggests that the short-term COVID-19 restrictions may have had limited impact on TB incidence.^15^

While the pandemic has unfolded differently according to contextual dimensions such as economic and health system capacities, asset organisation and deployment,^16^ it is imperative to leverage the substantial resources invested into health systems as part of the COVID-19 response to promote integrated care. For example, in the Central African Republic, the pandemic provided opportunities to reorganise health coordination; mobilise political capital for health systems strengthening; affirm the importance of community-focused responses; improve laboratory services; and invest more in the capacities and safety of healthcare workers (HCWs).^17^ Indeed, these lessons have been echoed in several LMICs in terms of promoting universal healthcare, including in South Africa,^18^ and have been promoted in framing challenges for LMICs in responding to TB and investing in the mental well-being of HCWs.^19 20^

The aim of this paper is to share our experiences in applying Learning Health Systems (LHS) thinking to understand the codevelopment of a health system strengthening intervention. The intervention aimed to improve TB literacy, screening, diagnosis and data, and HCW wellness, adapted to COVID-19, through the use of theory of change (ToC) strategising. By LHS, we refer to the ideal of ‘dynamic health ecosystems where scientific, social, technological, policy, legal and ethical dimensions are synergistically aligned to enable cycles of continuous learning and improvement to be routinised and embedded across the system’.^21^ It is thought that this data-driven approach can sustain and accelerate population health improvements through continuous innovation and adaptation.^22^ There is limited empirical literature on how LHS concepts can be operationalised, and there is urgency in better understanding how to build and sustain an LHS architecture to improve outcomes.^23^ The work presented there is part of the National Institute for Health and Care Research Health System Strengthening in sub-Sahara Africa (ASSET) programme, across multiple sites in Ethiopia, Sierra Leone, South Africa and Zimbabwe. With an overarching objective to promote person-centred, integrated and sustainable health system change for optimal TB care within the context of the COVID-19 pandemic, our subprogramme is focused in the Amajuba District in in the province of KwaZulu-Natal, South Africa.

The study was conceived as a sequential partially mixed methods study,^24^ comprising two work phases: (1) a diagnostic phase consisting of a scoping review, a situational analysis of a number of PHC facilities, formative semistructured interviews with key stakeholders, non-clinical observations in health facilities and (2) an intervention development, adaptation and piloting phase, involving the formulation of a ToC and codesigning and refining proposed interventions, piloting and evaluating codesigned interventions in PHC facilities.^25^ Outputs from the diagnostic phase are described elsewhere,^26 27^ and in this paper, focus is placed on the participatory formulation of a ToC and codesign process for the development of interventions which underpinned both phases of the research.

## Adopting an LHSs approach

This learning-oriented process is framed here using an LHS approach, which is an ideal organising principle for a data-driven response to a public health crisis such as COVID-19.^16 28^ LHS has several key features, including a designated network of multilevel stakeholders responsible for system design, operations and governance; common goals and a shared purpose held across these stakeholders; the generation of standardised approaches to quality care measures; leveraging technology to help facilitate knowledge flows between participants and the system; and a continued investment in trust, transparency and accountability among stakeholders.^23^ LHS approaches are seated on three key principles^29^:

1. Service users’ (ie, patients’) health and illness experiences should be captured as data and analysed.
2. Systems problems require systems solutions, meaning that organisational and cultural dimensions should be considered alongside individual-level dimensions of care.
3. Shorten the latency between knowledge generation and its application in real-world contexts.

**Figure 1:**
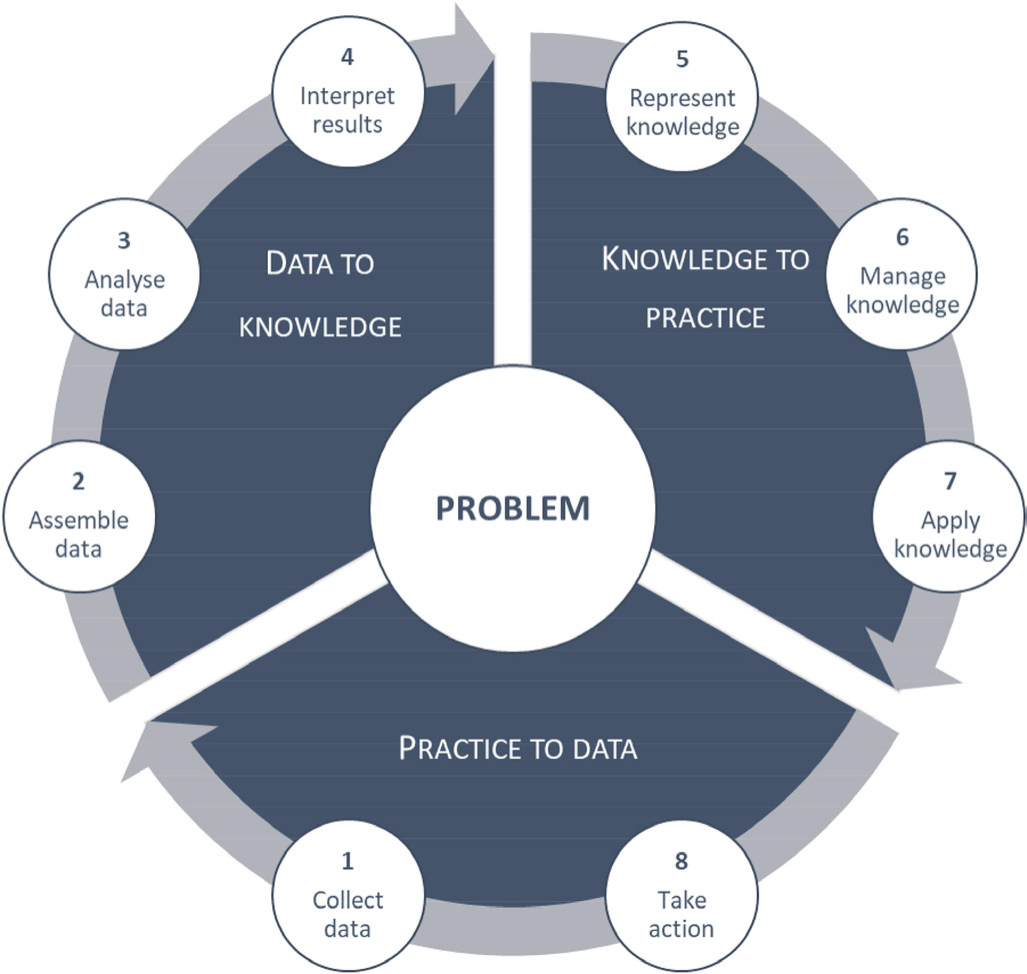
Learning health systems process of improvement.^30^

An LHS approach revolves around a continuous learning cycle of data generation and interpretation, and planning, coordinating and implementing appropriate interventions.^28^ A structured approach involves eight steps spread out during three cyclical, iterative phases: practice-to-data (P2D), data-to-knowledge (D2K) and knowledge-to-practice (K2P). The steps include (1) the collection of initial data; (2) assembling of data; (3) data analysis; (4) interpretation of the results; (5) representation of, (6) management of and (7) application of knowledge; and 8) taking action to change practice, setting the foundation for another cycle to be initiated.^30^ This process is illustrated in figure 1. At the core of the LHS process is a multistakeholder ‘learning community’, a coordinating group with shared interest in the problem under scrutiny. Appropriate representation in this learning community ensures that relevant data is collected during P2D, that collected data are analysed and interpreted from a range of perspectives during D2K, and that interventions are codeveloped and sustainable during the K2P phase.^29^ Following exploratory work in the P2D phase, participating health facilities are engaged to describe what practices are employed in relation to the central problem, analysed and interpreted (D2K phase) in terms of aggregated data across facilities, with some initial insights into how the identified problem could be overcome. This leads to a K2P processes, where data and recommendations are reviewed by the community of learning, and interventions are tailored to facilities, where, stimulated by the recommendations, new practices are adopted. Subsequently, a second P2D phase is initiated, where facility-level progress is documented and analysed, generating a next cycle.^31^

### P2D phase methods

A key aspect of the approach adopted is the initial, P2D phase, which offered a diagnosis of the challenges and opportunities for strengthening in the district. The methods of this particular phase has been described elsewhere,^27^ but are summarised here for context. Three data sources were used to develop an initial snapshot of TB care in the district. First, semistructured interviews were conducted with healthcare managers; clinical staff; private for-profit health service providers; and TB service users (the term ‘service user’ has its roots in South Africa’s National Health Act No 61 of 2003, which cemented a shift in legislation and policy from the passive, dependant term ‘patient’ to terminology that reflects someone who is entitled of basic human rights) in three PHC clinics and a regional hospital outpatient department. Second, direct, non-participant, semistructured observations were conducted within non-clinical areas of the four facilities. Researchers spent 2–3 hours in outside waiting areas, inside triage areas and waiting areas outside consultation rooms, filling out observation sheets—these were later used to draw process maps of service provider flow. Third, a document review was carried out. Policy and guidelines were reviewed to identify and highlight best practices for TB screening, testing, diagnosis, treatment and follow-up, and infection control. TB morbidity and mortality data were retrieved from the District Health Information System (DHIS) and reviewed for the reporting year of 2018–2019, which aided in highlighting facilities with higher burdens. This was further supported by a review of an internal TB mortality report of Amajuba. Facility-level routine TB data elements and indicators (monthly and quarterly breakdown of data elements according to the TB programme cascade, from screening, testing, to treatment, both total numbers and rates: people screened for TB symptoms; TB symptomatic clients testing positive; TB symptomatic clients with sputum sent; and TB clients started on treatment) from four preceding reporting quarters were extracted from the DHIS to further help identify possible health system bottlenecks.^27^

### ToC as planning tool

An LHS lens is applied to describe health system planning and implementation activities using ToC as a tool. ToC is an interactive, collaborative process that draws from a range of methods and data sources, describing how a programme intends to reach specific outcomes through a logical sequence of short-term and medium-term steps.^32^ An essentially pragmatic approach, it is not a once-off exercise but rather represents and steers a strategic process of engagement between stakeholders, open to regular modification as new data becomes available.^33^ Though there are many different ways in which ToC processes are approached, it usually results in the generation of graphic maps that illustrate how causal chains are expected to work, along with underlying assumptions that could influence succession towards overall outcomes.^32^ In this study, we drew from a range of quantitative and qualitative data sources, analysed and interpreted collectively in face to face and virtual workshops. The programme timeline, from conception to present, is presented in online supplemental file 1. A key output of this process is a package of codeveloped intervention strategies, designed to target key bottlenecks and piloted in a limited number of facilities before larger scaling up. The process leading to the development of this package is detailed here, drawing from the cyclical steps of an LHS approach.

10.1136/bmjgh-2022-009567.supp1 Supplementary data

### Patient and public involvement

Due to the focus of the study falling on collaborative working between a research team and healthcare management towards systems strengthening, service users and the public were not involved in the design, conduct or reporting of this study.

## Implementing the LHSs approach

### Establishing a learning community

Building on existing relationships with the district management through integrated mental healthcare initiatives, the research team met with key members of Amajuba District in 2018, after which initial data were collected on TB care bottlenecks. During August 2019, a 1-day workshop was held in a regional hospital in the district, where the key challenges and possible solutions to TB care in Amajuba District were discussed and interpreted among the research team and senior healthcare managers. Here, two important developments occurred: (1) An initial ToC map was developed on which to build going forward and (2) a collaborative coordinating structure was formalised where DoH stakeholders and the research team together formed a learning community. This Learning Community was engaged in the refinement of the proposed challenges, identifying poor TB outcomes and discussing possible causes, as well as possible solutions to these problematic causal processes. Prior to piloting of proposed interventions, the ToC and associated objectives were revised to assist in supporting efforts to fighting COVID-19. This was especially pressing, after initial reports surfaced about the programmatic effects that the pandemic had on other priority programmes such as TB and the psychosocial well-being of HCWs. Little to no intervention work could be conducted during the first wave of COVID-19 infections, though the Learning Community team continued to communicate virtually. During standing weekly meetings, the Learning Community adapted the original, TB-focused ToC to focus on newly identified challenges, namely the integration of COVID-19 responses with TB, and addressing the psychosocial burden of the pandemic on HCWs.

### Practice-to-data

From 2018 to 2019, prior to the COVID-pandemic, a diagnostic work package was carried out that formed the P2D phase. The focus in the diagnostic phase was to collect data that would allow us to generate theoretical understanding of the contextual determinants of TB care. This required building relationships and trust with staff over time, and sensitively negotiating the use of formal research methods of interviews and observations within everyday clinic settings. These interactions provided critical insights in themselves. Requests to interview or observe practice, or informal discussions with staff revealed concerns over individual accountability and anonymity which located these encounters within the wider political, institutional and sociocultural context of TB. We then reflected on these experiences in research team meetings, deepening our understanding and allowing us to formulate a preliminary hypothesis: TB treatment outcomes can be improved in the district by improving (1) service users’ experiences of care, (2) the clinical management of TB and comorbid TB and depression, and (3) facility-level TB infection control. This improvement can be achieved through a codeveloped intervention package that leverages clinical training and mentorship, clinical communication skills training, and stigma reduction and self-care strategies for service users.

**Data to knowledge**

**Figure 2:**
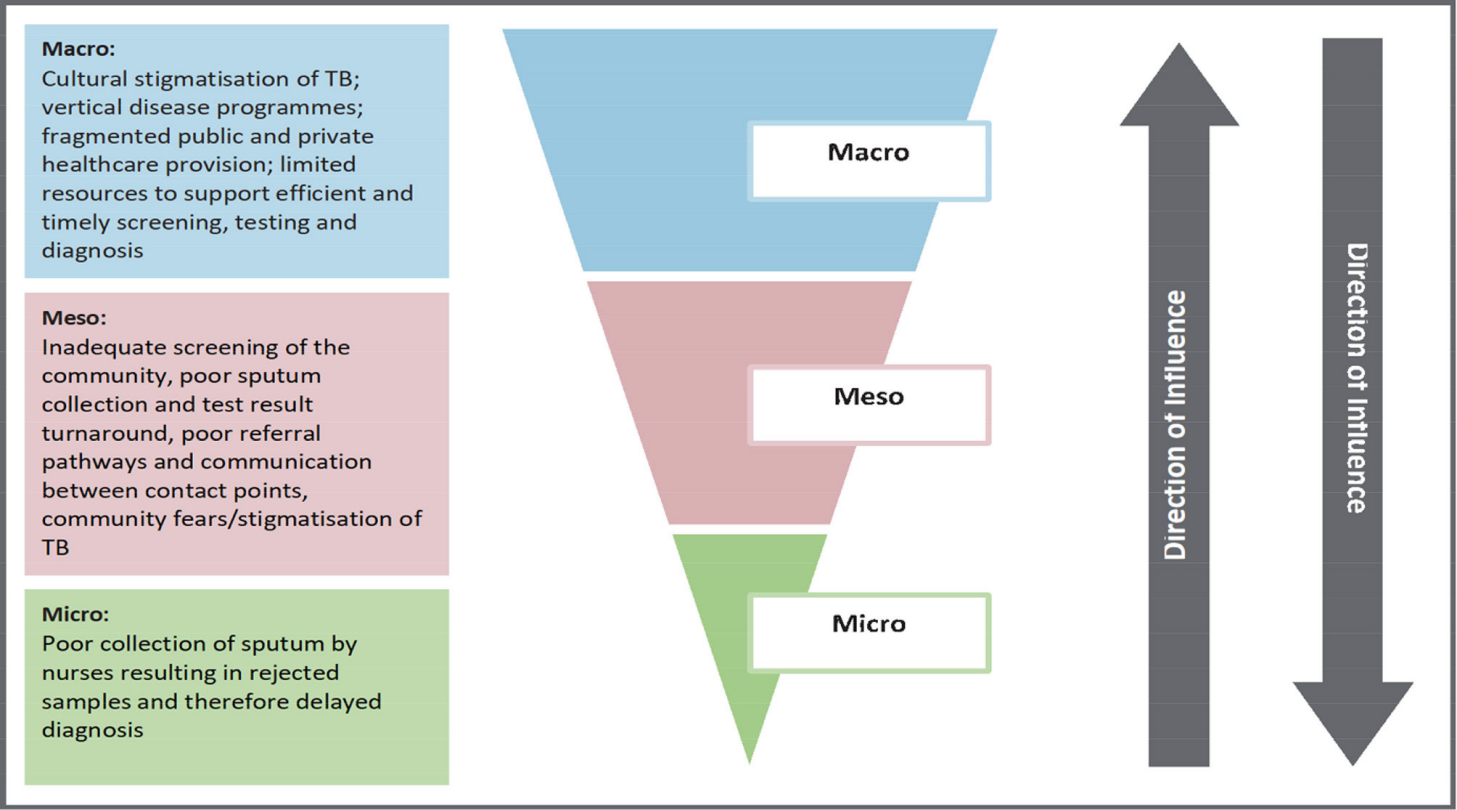
Findings on delayed diagnosis at community/clinic level. TB, tuberculosis.

Following the diagnostic phase of the project, data were assembled, thematically analysed and interpreted using the Context and Implementation of Complex Interventions framework.^34^ Accordingly, several contextual factors were identified that influence TB care in The District. For example, as illustrated in figure 2, factors that delay TB diagnosis included macrolevel cultural stigma against TB, verticalisation of programmes and fragmentation between public and private spheres; mesolevel inadequate community screening and poor referral pathways and microlevel poor sputum collection practices.

As mentioned earlier, these findings were presented and cointerpreted at the 1-day workshop in August 2019, which resulted in the development of a first draft ToC map. In this configuration, the findings were assembled into the main levels of the TB care cascade, namely, Early Identification, Diagnosis, Early Treatment Initiation and Treatment Adherence and Support. Furthermore, key problems and possible solutions were stratified according to community and PHC facility level. Importantly, a key guidance during this early period of ToC development focused on the urgency of reducing substantial TB mortality rates in the District. Subsequently, the main, long-term outcome that was decided among the group was to reduce deaths in the District from drug-sensitive TB. Four factors were identified that could help to achieve this goal, namely to improve TB case finding; to improve early diagnosis; to improve treatment monitoring; and to improve education and psychosocial support among service user.

The internal logic of this initial draft map assumed that if people with TB symptoms are identified earlier and referred for diagnosis; if people who have been referred for diagnosis and were found to be positive for TB are earlier initiated on treatment; and if people on treatment receive adequate psychosocial and treatment adherence support; then, people’s TB disease severity would be curtailed and their clinical prognosis would improve, leading to a reduction in mortality rates.

During refinement of the ToC draft, the Learning Community collectively decided that more exploratory data was required to better understand the causal linkages described in the ToC. This led to a second round of data gathering, where additional facilities were included to conduct interviews with people presumptive with TB. At a 1-day workshop in December 2019, these additional insights were assembled and interpreted within the Learning Community to further revise the ToC map, which, following several iterations, ultimately focused on the following priority objectives: (1) poor literacy among service users; (2) inaccurate TB screening in PHC facilities; (3) poor sputum sample quality and (4) poor data quality. A key consideration in this amendment was time and resources available to reach appropriate targets for the ASSET programme. At this stage, towards the end of 2019, an intervention package was designed to address these identified challenges. The intervention package included a three-pronged approach:

1. A training package for HCWs, focusing on TB screening and diagnosis (target beneficiaries: PHC HCWs).
2. Educational posters and leaflets that provide targeted information in local languages about TB prevention, identification and management (target beneficiaries: Service users visiting PHC clinics; PHC HCWs).
3. Mentoring support in implementing changes and sustaining new and adapted practices in screening, sputum sample collection and data management (target beneficiaries: PHC HCWs and management).

### Knowledge to practice

Once the intervention package was agreed among the Learning Community, materials were developed and planning was initiated to conduct pilot training in selected facilities towards the start of 2020. These activities—which initiated the K2P phase—started in one pilot facility, just before the first COVID-19 lockdown period. By February 2020, the pandemic spread in South Africa to such an extent to raise serious concern, and by March the first of several national lockdowns was put in place, thereby freezing all research and development activities.

**Figure 3:**
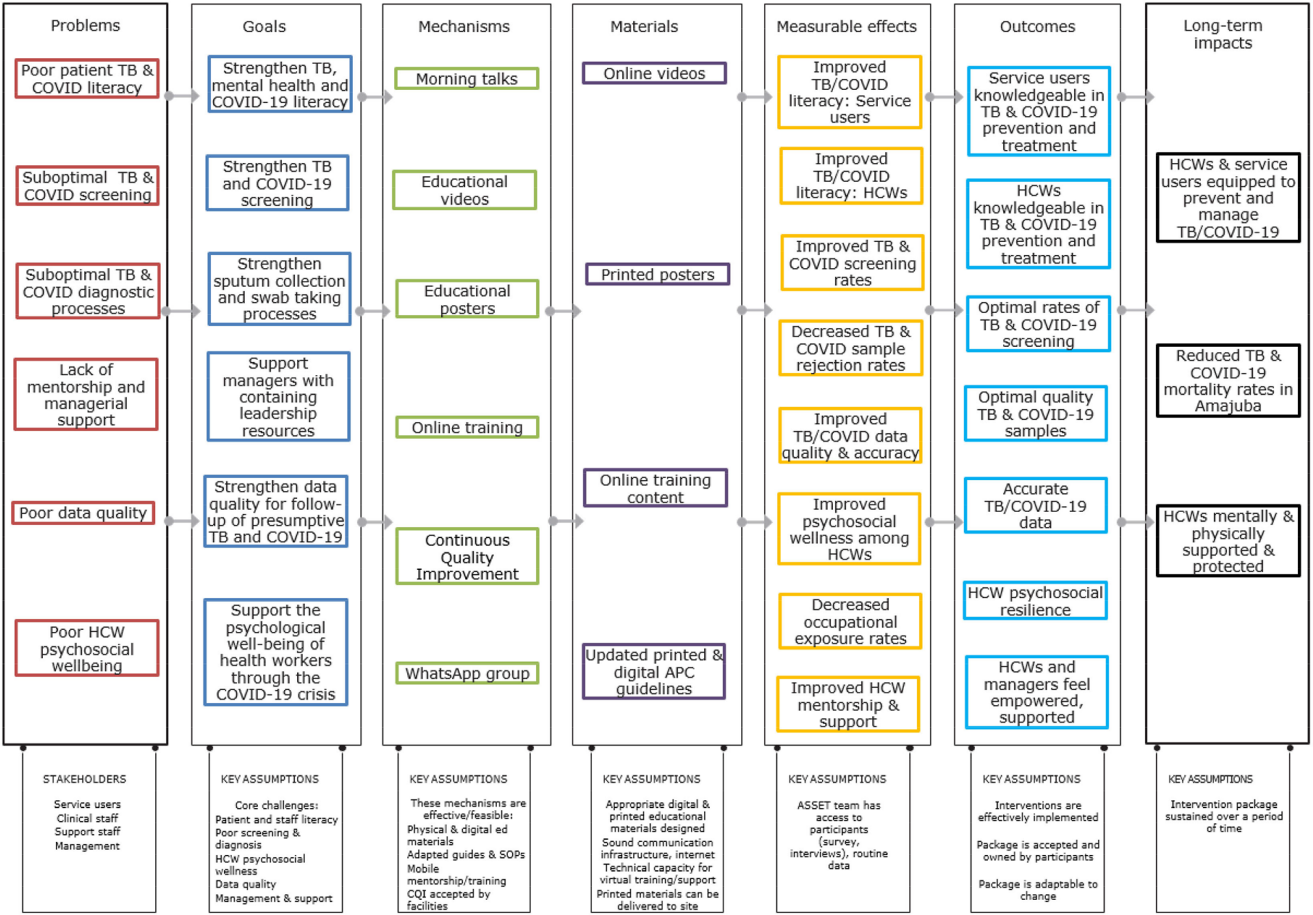
ToC map for the integration and strengthening of COVID-19 and TB programmes on PHC level. HCWs, healthcare workers; PHC, primary healthcare; TB, tuberculosis; ToC, theory of change.

Given the new contexts and new set of challenges, it was necessary to first return briefly to the P2D phase. A site visit of two PHC clinics was undertaken by members of the ASSET team, which included observations of patient pathways in terms of TB and COVID-19, as well as interviews with facility and district staff. The data were rapidly transcribed and analysed thematically, and included in learning community discussions on an adapted intervention package. Accordingly, key areas of concern were identified, including poor literacy among service users on TB and COVID-19; suboptimal screening for TB and COVID-19 in facilities; suboptimal diagnostic processes for TB and COVID-19 in facilities; poor TB and COVID-19 data quality; substantial strain on the mental well-being of staff; poor infection control practices; and suboptimal mentorship in dealing with a rapidly changing emerging disease. The Learning Community team subsequently revised the ToC (figure 3) and agreed that the project fixed its focus on the following objectives:

- Strengthen TB, mental health and COVID-19 literacy.
- Strengthen sputum collection and swab taking processes.
- Strengthen TB and COVID-19 screening.
- Strengthen managers’ capacity to contain anxieties of staff in the context of the additional stressors imposed on them by the COVID-19 pandemic.
- Strengthen data quality for follow-up of presumptive TB and COVID-19.
- Support the psychological well-being of health workers through the COVID-19 crisis.

**Figure 4:**
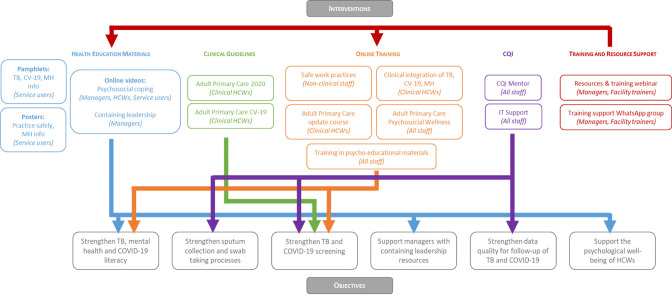
Relation between codeveloped interventions and objectives. CQI, continuous quality improvement; HCWs, healthcare workers; TB, tuberculosis.

As depicted in figure 4, five intervention packages were developed to address the objectives generated in the ToC process. Health education materials that included pamphlets and posters (digital and hard copy) were developed to increase service user and HCW literacy in TB, COVID-19, and mental health, as well as to promote safe practices in facilities.^35^ Online videos were developed to support psychosocial coping among managers, HCWs and services users, as well as to develop capacitate managers with skills to contain staff anxieties.^36^ This was generated in partnership with a sister programme—the Southern African Consortium for Mental Health Integration (S-MhINT).^37^ Updated Adult Primary Care (APC) clinical guidelines—nationally endorsed PHC guidelines—were provided to facilities, as well as APC COVID-19 guidelines.^38^ The distribution of these materials was supported by various online training initiatives, including courses for non-clinical staff on safe work practices; accompanying training on APC guidelines for clinical staff; a course on psychosocial well-being and orientation on psychoeducational materials; and training on the clinical integration of TB, COVID-19 and mental health on PHC level. In addition to training HCWs, an additional step focuses on the development of facility-based trainers, who can decentralise training facilitation from Cape Town-based facilitators to include staff members who can help support and drive online training participation. Details of the intervention are provided in online supplemental file 2, presented in terms of the Template for Intervention Description and Replication checklist.^39^

10.1136/bmjgh-2022-009567.supp2 Supplementary data

These educational and training resources were further supported by the adoption of a continuous quality improvement (CQI) approach. In partnership with S-MhINT and the DoH, a CQI mentor was recruited, trained and positioned in the District. The CQI coordinator has a dedicated office space in the district DoH offices, thereby creating a more direct line of support. Furthermore, the CQI mentor works closely with PHC facilities, to work with staff to, by using tools such as Plan–Do–Study–Act, identify bottlenecks and challenges related to data management, diagnostic sample collection, screening and infection control for TB and COVID-19, and codeveloping solutions tailored to each facility. A good example of an unforeseen challenge emerged in terms of technical capability and resources among some staff to be able to connect to online training platforms. In order to bridge this gap, a technical assistant was employed to provide hands-on support to staff members during online training sessions. A related challenge was the cost of data, which was bridged by enlisting the services of a third-party business to render all online training and educational content data-free, thereby removing a significant barrier in accessing resources. Finally, the distribution of health education resources and the supporting of online training was facilitated by an online webinar for managers and facility trainers, as well as through the establishment of a training support WhatsApp group.

#### Next steps

**Figure 5:**
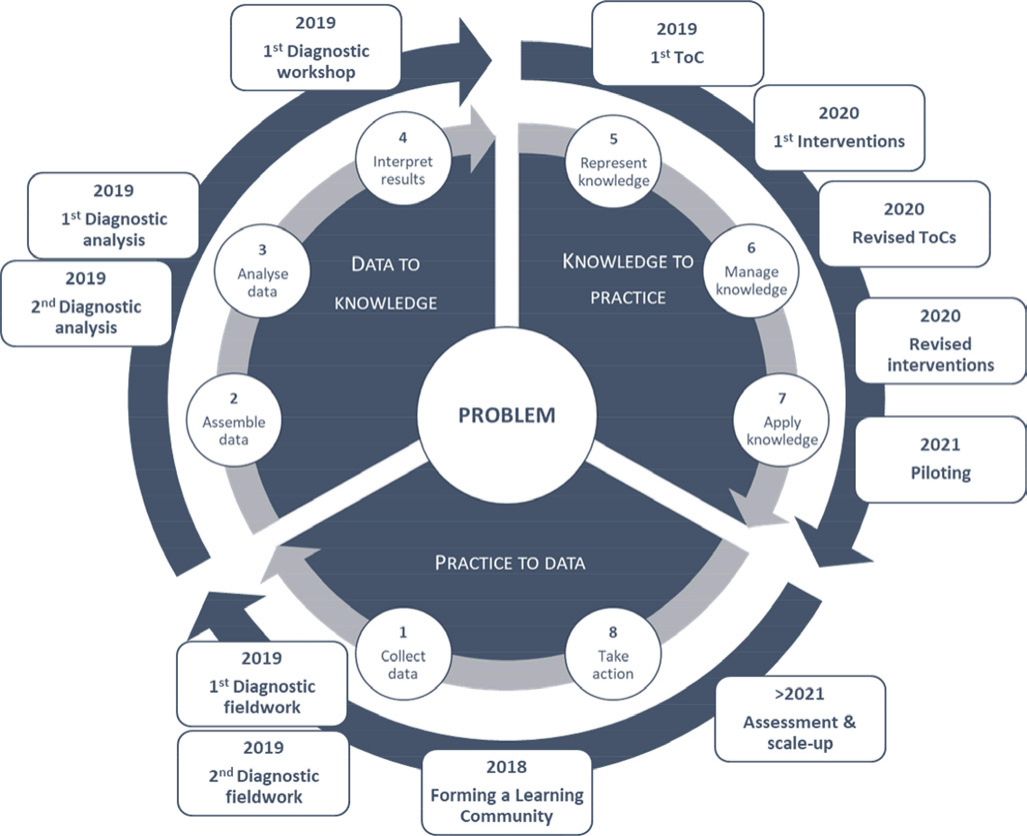
Programme timeline as interpreted through an LHS approach. LHS, learning health systems; ToCs, theory of changes.

Following the development of the revised intervention package in the K2P phase, piloting was initiated in a limited number of facilities. As illustrated in figure 5, this is followed by wider implementation, which is monitored in weekly learning community team meetings. In addition, process evaluation data, collected at different time points, are analysed along with pre–post survey data and routine indicators, will form the basis of evaluation of the effectiveness of the intervention package on key outcome measures.

## Lessons learnt

The impact of the COVID-19 pandemic on health system functioning has been profound, the full range of proximal and distal effects of which are yet to be fully grasped. In the context of this pandemic, LHS approaches has shown great potential for improving system preparedness through collaborative learning and technical support and data-driven decision-making.^16^ Four main lessons have been gleaned from our experience in adopting an LHS approach in a South African district, which are (1) the importance of building and sustaining relationships, (2) the utility of colearning, coproduction and adaptive capacity, (3) the centrality of theory-driven systems strengthening and (4) reflections on LHS as a framework.

### The importance of building and sustaining relationships

Our first lesson, and certainly the most obvious and well known, is the importance of building and sustaining relationships of collaboration, trust and mutual support, between researchers, policy makers, managers and front-line workers—not only in the beginning phases of a programme, but throughout its trajectory. Established relationships and collaborative structures are vital starting points for LHS cycles to be initiated and sustained over time.^40^ In this vein, our programme’s sustainability was significantly elevated by a pre-established relationship between the research team and district stakeholders, which facilitated more resilience in responding to emerging challenges. In the emergency contexts of COVID-19, resources needed to be maximised more than ever. A participatory, flexible approach was therefore especially useful in bringing together and sharing resources across government and academic sectors, as well as across different programmes with different funders. The value of collaboration was key in helping the ASSET programme to adapt to rapidly changing contexts. In this vein, a case is to be made—based on our experiences relayed here—that more investment is required into building relationships, in addition to developing programme content. Investing in the medium-to-long term nature of building and sustaining trust and cooperation between researcher and local partners, rather than a myopic focus on project lifecycles, has important consequences for how health system strengthening initiatives are funded and supported in global health.^41^

### The utility of colearning, coproduction and adaptive capacity

The second lesson that emerged from our experience relates to the importance of colearning. It has become increasingly critical for health systems—especially ones in LMICs—to embrace collaborative and dynamic forms of learning.^22^ As mentioned earlier, a key strategy in LHS is to speed up knowledge translation through colearning and coproduction. More traditional, static methods of knowledge dissemination with limited uptake, for instance, policy briefs, are simply not effective in response to a complex and rapidly changing environment.^42^ The creation of a space where mutual learning can occur, and then result in the coproduction of potential solutions, was a valuable tool in adapting to the uncertainties raised by COVID-19. There is a growing body of work describing the benefits of collaboration, partnership and coproduction in health systems, particularly in LMIC settings, where local ownership is a critical factor in determining successful outcomes.^43^ A central expectation of coproduction—the explicit inclusion of stakeholders in the development of the agenda, design, implementation, interpretation and dissemination of research—is to generate findings that are relevant, useable, useful and used.^44 45^ Sustained coproduction and learning are further intertwined with trust, accountability and mutual benefit, particularly in fragmented health systems.^23^ While the ideal of coproduction has received increasing attention during the past decade, the urgency to adopt the approach in real-life settings have been propelled forward substantially by COVID-19. While some coproduction cases are embedded in large, macro structures, for instance, Thailand’s ‘triangle that moves the mountain’ initiative (a framework relying on three power poles of collaboration, between a government sector, a knowledge sector and a people’s sector),^46^ others are on a much smaller scale—our work falls in the latter.

Our experience highlights the need for a fair degree of adaptive capacity—not only in the health system, but also in terms of research programmes. Adaptive capacity, meaning ‘the capacity to make intentional incremental adjustments in anticipation of or in response to change, in ways that create more flexibility in the future’^47^ is a key dimension of resilience in the face of external shocks such as COVID-19. Lessons from the 2014–2016 Ebola outbreaks underlined the necessity of resilience and the capacity to effectively anticipate and deal with uncertainties.^48^ Specifically, stakeholders in health systems strengthening need to anticipate disruptions and opportunities, be able to monitor system processes, respond to immediate demands, and learn from experience.^49^ The regular engagement in our collaborative learning community team facilitated a sharing of valuable front-line knowledge as the impacts of COVID-19 emerged, and allowed for a more rapid and effective response to identified gaps. Adaptations to TB educational materials to also include COVID-19, and the development of platforms to address psychosocial wellness among overburdened staff are examples of this. The latter was particularly critical, given our findings that suggest high levels of COVID-19-related mental strain among HCWs, and a substantial proportion of managers being in known high-risk groups to contract the disease.^50^ Managing emergent anxieties associated with healthcare work remains a central concern. Accordingly, the programme was able to draw from ‘situated resilience’, adaptations to front-line facing and operational elements of care.^51^ Another major advantage in this experience was the ability of our programme to shift and change its objectives from the original proposal to adapt to emergency circumstances. Our ASSET work package also benefited from substantial responsiveness from the S-MhINT programme, by partnering in supporting a CQI mentor and to develop psychosocial wellness materials towards achieving common goals. The ability of a programme to adapt to emerging challenges and to draw from additional resources are essential considerations in health systems strengthening. Nonetheless, purposeful and sustained investment in developing LHS are rare, and health systems strengthening unfortunately occurs inconsistently and often in response to crisis. As concluded in a recent paper, ‘learning investments are not ‘quick fixes’. They do not guarantee rapid or predictable returns. They require the patience and perseverance to nurture human capital for learning, and to conceive and grow learning institutions’.^22^

### The centrality of theory-driven systems strengthening

Our third lesson relates to the need for health system strengthening activities to be theory-driven. While an LHS approach offers flexibility and adaptive capacity, there is an inherent danger of the work becoming problem-driven and reactionary, undermining strategic operationalisation of lessons and rigorous scientific process. By keeping the programme theory at the heart of the enterprise, its lessons become transferable and our understanding on how to strengthen systems improves.^52–54^ Theory in this sense does not mean fixed and unchanging, but rather a roadmap that constantly requires updating and refinement. As previous similar initiatives have shown, coproduction processes within an LHS frame are very much built on key questions about collective sense-making, the nature of evidence and evidence generation, and analysing and theorising such evidence.^55^ In this paper, we have demonstrated how the application of ToC can help facilitate LHS cycles by offering guidance on how the programme theory evolves over time. Beyond ToC’s apparent linear logic, examples from Bangladesh, Uganda and India illustrate how ToC can be used as a valuable strategising tool to achieve consensus in a cyclical way.^52^ In addition to its traditional application as a monitoring and evaluation tool, the ToC double-loop process is designed to facilitate organisational learning, through critical, collective reflection.^56^ This was particularly an important communication and strategising platform within our learning community engagement. Moreover, ToC is particularly suited to a systems approach, rather than vertical, disease-focused, project-oriented approaches^57^—this was vital in adapting our ASSET programme to the pandemic and integrating COVID-19 with TB care to promote more person-centredness.

### Reflections on LHS as a framework

A final, brief point to be made relates to comments on the LHS as a framework. The approach has fairly recently been formalised, and, to our knowledge, is yet to be rigorously assessed as a pragmatic tool. Nonetheless, our experience suggests that the LHS as a framework offers enough flexibility in its three broad phases to incorporate localised adaptations to the guidelines as originally proposed. A potential area in the framework that could be expanded on is the role of power, given the central concept of coworking and collaboration. Highlighting power more explicitly, with potential strategies to manage different structures, networks and relations of power, might provide helpful guidance for people working in pluralistic health systems such as South Africa’s, often fraught by politics and competing priorities. Further, as noted in the previous paragraph, there might be space for a more central inclusion of theory as an anchoring mechanism of the highly dynamic LHS process. In ASSET, we adopted ToC to help mapping out the programme theory as well as to monitoring changes made in response to new data and needs that might emerge during health systems strengthening. However, integrating a ToC approach within the LHS cycles might help strengthen the framework and allow for a more systematic generation of intervention options and enhance the transferability of lessons to other regions.

## Conclusion

Our experiences in closely collaborating and codeveloping solutions with a district health management team in South Africa, amidst the chaos and uncertainty of a global pandemic, illustrate the benefits of an LHS approach, particularly using ToC as a strategic tool with which to disentangle the complexities of the local health system. The pandemic conditions reiterated how critical policy decision-making is amidst emerging evidence,^58^ and traditional cycles of research and evidence generation has become impractical and flawed. Importantly, coproduction and colearning facilitates the development of more medium to long-term resilience in embedding health systems researchers in the ecosystem of decision-making,^59^ laying a foundation for ongoing collaboration and health systems strengthening beyond crisis.

## Data Availability

Data are available on reasonable request. Data are available on request from the corresponding author.
