# Supplementary figures and images for: Applying learning health systems thinking in codeveloping integrated tuberculosis interventions in the contexts of COVID-19
Source: BMJ Glob Health. 2022 Oct 31;7(10):e009567. doi: 10.1136/bmjgh-2022-009567 (PMC9627575; doi:10.1136/bmjgh-2022-009567)

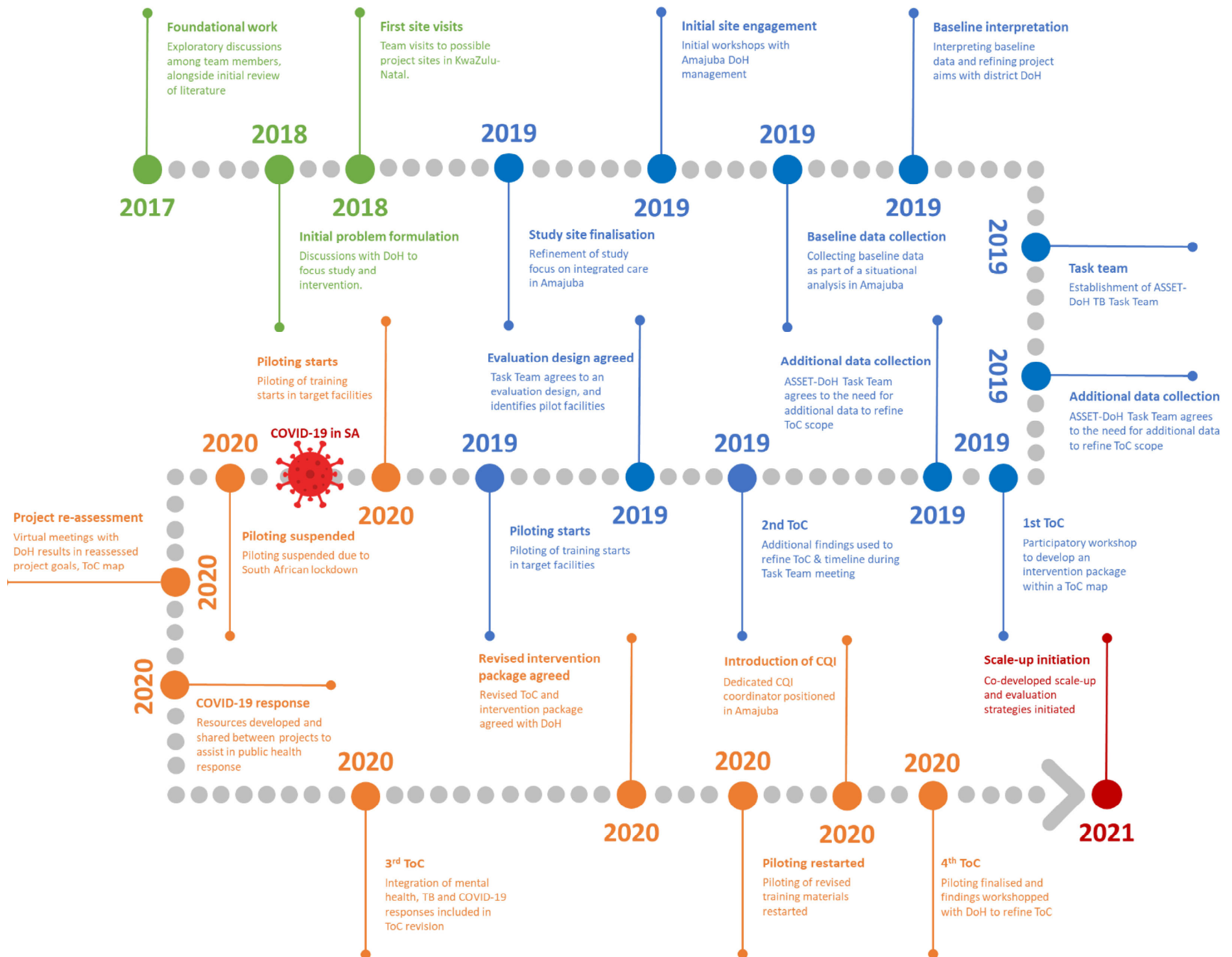

Supplement: Supplementary data [file bmjgh-2022-009567supp001.pdf]
