# Supplementary material for: Applying learning health systems thinking in codeveloping integrated tuberculosis interventions in the contexts of COVID-19
Source: BMJ Glob Health. 2022 Oct 31;7(10):e009567. doi: 10.1136/bmjgh-2022-009567 (PMC9627575; doi:10.1136/bmjgh-2022-009567)
Supplement: Supplementary data [file bmjgh-2022-009567supp002.pdf]

TIDieR framework:ASSET
09 May 2022

TIDieR framework: ASSET hybrid training intervention

**1. Brief name**	Integration of TB and COVID-19 hybrid training intervention
**2. Why**	The hybrid training intervention package was initially developed to address the delayed diagnosis and consequent death of people with TB in the high burden TB district of Amajuba. Some of the contributing factors identified in the situational analysis included an interaction between verticalized TB care provision, limited resources; poor quality of TB screening, sputum collection and data capturing, and patient level challenges such as employment demands, discontinuity of information and limited support on treatment. With the advent of COVID-19 and its shared symptoms with TB, coupled with the reduction in TB notifications, the intervention required adaptation to integrate screening and testing for these conditions. Due to limitations of lockdown regulations on in person training, the training was developed on an online platform for accessibility.
**3. What - materials**	The training content was developed on an online platform and was divided into 4 sessions. The content of the training included: i) An introduction and overview of the research and intervention, as well as an introduction to the APC COVID-19 guidance job aid. ii)Due to the level of stress regarding the COVID-19 pandemic this was addressed in content covering managing health worker exposure to COVID-19, how to practice safely using correct PPE, coping with occupational stress and caring for mental health. iii) COVID-19 and TB screening and diagnosis using case scenarios. iv) the integration of COVID-19 and TB management, including specimen collection.<br><br>The case based model of APC training (ref training paper) used in the in person training was adapted for the online space. Instead of a facilitator guiding the navigation of the APC clinical tool/APC COVID-19 job aid using a case scenario formatted in a paper based template, the online training was structured as a quiz with the case scenario information presented, a question posed, and multiple answers optional to select. The user would select the answer/s they thought correct based on their reference to the APC clinical tool/APC COVID-19 job aid. The correct answers are then given with an explanation as well as an image of where to find the answer in APC clinical tool/APC COVID-19 job aid.<br><br>**Resources that all facilities received:**<br>• APC 2020 (printed and eBook)<br>• APC COVID-19 guidance (printed and eBook)<br>• TB, COVID-19 and Mental Health information pamphlets<br>• Practice safely and Mental health posters<br>• Online self help psychosocial coping skills building videos for managers, providers and patients/community<br>• Integrated TB and COVID-19 clinical online training course, data free<br>• Introduction to COVID-19: how to work safely in a health facility - Online course for non-medical staff, data free<br>• APC 2020 update online training course, data free<br><br>**The 10 intervention facilities received this additional support:**

TIDieR framework:ASSET
09 May 2022

	• Managers and Facility trainers attended a webinar to be orientated to the resources and training platform<br>• Staff had access to a whatsapp group for training support
**4. What - procedures**	**Date** / **Activity**

**Date**	**Activity**
07 Aug 2020	300 APC clinical tools and 300 APC COVID-19 job aids delivered in Amajuba for all clinics
September 2020	Bonisiwe was mobilised to support ASSET
10 September 2020	Delivery of posters and pamphlets to Amajuba
23 September 2020	Amajuba Managers session - orientation to ASSET for all facility managers, with access to resources listed above
23 September 2020	300 Updated APC COVID-19 job aids printed and delivered to Amajuba to replace outdated guidance.
?01 October 2020	Sanah started working
14 October 2020	Blended ASSET training orientation for Pilot Facility Manager's and Facility Trainers (Webinar with technical support)
26 October 2020	10 Devices (Tablets) delivered to the Amajuba District Office for distribution to each intervention facility
17 November 2020	feedback session with Rosary clinic (Pilot clinic)
17-18 November 2020	Intervention facility Manager and FT training<br>8 clinics in 2 sessions
19 November 2020	feedback session with Newcastle clinic (Pilot clinic)
March 2021	Feedback sessions with the intervention clinics
3 Nov 2021	Memo for protected time sent out from District

5a. Who provided

23 September 2020	Amajuba Managers session - orientation to ASSET	Christy-Joy Ras on webinar, Inge Peterson and Andre Janse van Rensberg in person
14 October 2020	Pilot Facility Manager and Facility Trainer (FT) training	Christy-Joy Ras, Lauren Anderson<br>Bonisiwe Skosana for tech support<br>Nomathemba Mazibuko (District training manger) in person

TIDieR framework:ASSET
09 May 2022

	17 November 2020	feedback session with Rosary clinic (Pilot clinic)	Christy-Joy Ras, Robyn Curran
	17-18 November 2020	Intervention facility Manager and FT training 8 clinics in 2 sessions	Christy-Joy Ras, Lauren Anderson<br>Bonisiwe Skosana and Sanah Bucibo for tech support<br>Nomathemba Mazibuko in person
	19 November 2020	feedback session with Newcastle clinic (Pilot clinic)	Christy-Joy Ras, Robyn Curran
	March 2021	Feedback sessions with the intervention clinics	Christy-Joy Ras, Robyn Curran
**5b. Who received**	23 September 2020	Amajuba Managers session - orientation to ASSET	Facility Managers from the district clinics
	14 October 2020	Pilot Facility Manager and Facility Trainer (FT) training	Facility Manager and Facility Trainer from Rosary and Newcastle clinic
	17-18 November 2020	Intervention Facility Manager(FM) and FT training<br>8 clinics in 2 sessions	17th Nov: FM Osizweni 2<br>CNP Osizweni 2<br>CNP Charlestown<br>FM Osizweni 3<br>18th Nov: FM Naasfarm<br>OM Nellies Farm<br>OM Stafford<br>OM Madadeni Hosp<br>OM Madadeni 7
**5c. Who benefits**	1. Clinicians – training equips them with information to manage patients as well as occupational stress management<br>2. Patients – those presenting with undiagnosed TB		
**6. How**	The Intervention was introduced to all facility managers in the district, who were given all the same resources – hard copies of the guidance and links to the online training courses to disseminate to their staff.<br><br>A webinar was the held with the Facility Managers of the 2 pilot facilities, who were together in a room with appropriate social distancing and masks. The trainers were online and projected up for the group to see. The content of this training was orientating the pilot facility managers and trainers to how to access the online platform, as well as how to navigate it. The first online session content was completed with them, supported by a team member in the room who could manage technical challenges. In this session they were mandated to return to their facilities and cascade what they had experienced with their colleagues. A whatsapp group was set up for support and follow-up.<br><br>A Quality Improvement (QI) Mentor was hired to be based in Amajuba District at this point.		

TIDieR framework:ASSET
09 May 2022

	A month later the same webinar training was run for the eight intervention facilities in two separate sessions. Again there was technical support present to help the participants create an account on the online training platform, and navigate through the content of the first session. A whatsapp group was set up for each group.<br><br>The cascade back to the intervention facilities by those who attended the training was sporadic and poor, so the QI mentor began to offer support on her clinic visits. This greatly increased the uptake of the online training, and so this support structure was implemented in all facilities to get staff engaged with the online training.<br><br>Feedback sessions with the pilot facilities were run a month after their training. The feedback sessions with the other eight clinics were only able to occur four months after the training.<br><br>A 'Learning session' was planned and held with relevant representatives from the District and facilities to reflect on different aspects of the intervention. At this forum the online learning was topic for discussion. One of the main barriers that was expressed there was a lack of protected learning time at work to be able to progress through the course.<br><br>As it was not sustainable for the QI mentor to be the online support champion for all the facilities, the implementation model for the online training was revised. In collaboration with the District it was decided that instead of training Facility Trainers as previously were required to deliver APC training, APC online champions would be trained to support colleagues to access online training, deal with technical problems, and thereafter organise small group sessions where people could either progress together through the training content, each on their own device, or complete at their own pace, but in a group with access to others for discussion and shared learning.<br><br>The first training programme for APC online champions was run in September 2021, in a face-to-face training, where 27 people attended from across the district.<br><br>Support was given to the District to compile a memo to send out for protected onsite training time. This was circulated in support of the APC online champions arranging onsite, in-service time for nurses to gather and progress through the online learning content on their devices.<br><br>A Facility level progress report, showing staff progress through the online training content was also designed. Included on this report was facility training coverage, which took baseline clinic staff data collected by an onsite audit, cleaned and entered into the database, as the denominator, and calculates training coverage with the number on staff registered online as the numerator. This report has been generated every second week for distribution by the QI mentor via whatsapp to each APC online champion to be able to track progress of staff towards completion. The ability to monitor training progress internally due to online training has transformed the way support can be targeted.

TIDieR framework:ASSET
09 May 2022

7. Where	Due to limited in person contact and travel restrictions, training had to take place in a blended format.<br>The first training for all district managers occurred during a low period of transmission at the District office, with all social distancing and mask wearing protocol in place. The UKZN team Inge Peterson and Andre Janse van Rensburg were able to support in person with the Cape Town based trainer, Christy-Joy Ras, on a webinar.<br><br>The training for the pilot and intervention facility Managers and facility Trainers took the same format, with onsite support from the UKZN staff based in Amajuba, and the Cape Town based trainers on a webinar.<br><br>The QI mentor offered in person support where possible, and via whatsapp during high risk periods.<br><br>The Learning Session took place at a local venue in Amajuba during a low risk time, and was facilitated in person by the UKZN and UCT teams, with a good turnout from the district.
8. When and how much	The training for all the managers was a once off, half day programme.<br>The training for the pilot and intervention facilities was also a once off, half day programme.
9. Tailoring	The learning from this intervention was vast and has led to the development of a blended learning cascade model. The parts of the intervention that could be tailored are as follows:<br>• The devices that were provided to the ten intervention facilities were not necessary. The staff used their own devices as the training did not cost their data.<br>• The need for technical support was greater than anticipated and has to be provided for the successful uptake of training.<br>• A specific role for an Online Champion to take up the responsibility of providing a learning environment (orientating staff to the online training, dealing with registration challenges, and then maintaining training momentum by organising small group sessions of learning), has been created. This was not planned for, but tailored to meet the need that emerged.<br>• The stress levels of the nurses due to COVID-19 were very high. This prompted the development of an online wellness resource for nurses to access information and resources to support them in various challenges that they were facing.<br>• Facility Level training progress reports were designed to be able to support the online champions to monitor and support their colleagues complete training.
10. Modifications	Listed here are the modifications that were made to the training:<br>• An Online Champion Training programme<br>• An Online Champion Manual was designed which includes an instruction manual of how to assist people to set up an email address, create and save passwords, create an online profile and navigate the online platform.

TIDieR framework:ASSET
09 May 2022

	• Facility Level progress reports in pdf format to be able to send out via whatsapp
